# The genetic architecture of Plakophilin 2 cardiomyopathy

**DOI:** 10.1038/s41436-021-01233-7

**Published:** 2021-06-12

**Authors:** Annika M. Dries, Anna Kirillova, Chloe M. Reuter, John Garcia, Hana Zouk, Megan Hawley, Brittney Murray, Crystal Tichnell, Kalliopi Pilichou, Alexandros Protonotarios, Argelia Medeiros-Domingo, Melissa A. Kelly, Aris Baras, Jodie Ingles, Christopher Semsarian, Barbara Bauce, Rudy Celeghin, Cristina Basso, Jan D. H. Jongbloed, Robert L. Nussbaum, Birgit Funke, Marina Cerrone, Luisa Mestroni, Matthew R. G. Taylor, Gianfranco Sinagra, Marco Merlo, Ardan M. Saguner, Perry M. Elliott, Petros Syrris, J. Peter van Tintelen, Regeneron Genetics Center, Cynthia A. James, Christopher M. Haggerty, Victoria N. Parikh

**Affiliations:** 1 Stanford Center for Inherited Cardiovascular Disease, Division of Cardiovascular Medicine, Department of Medicine, Stanford University School of Medicine, Stanford, CA USA; 2 Invitae, Inc, San Francisco, CA USA; 3 Laboratory for Molecular Medicine, Mass General Brigham Personalized Medicine, Cambridge, MA USA; 4 Dept. Pathology, Massachusetts General Hospital, Harvard Medical School, Boston, MA USA; 5 Division of Cardiology, Department of Medicine, Johns Hopkins University, Baltimore, MD USA; 6 Dept. of Cardiac-Thoracic-Vascular Sciences and Public Health, University of Padua, Padua, Italy; 7 Centre for Heart Muscle Disease, Institute of Cardiovascular Science, University College London, London, UK; 8 SwissDNALysis-Cardiogenetics, Dübendorf Switzerland, Zurich, Switzerland; 9 Geisinger, Danville, PA USA; 10 Regeneron Genetics Center, Tarrytown, NY USA; 11 Cardio Genomics Program at Centenary Institute, The University of Sydney, Sydney, Australia; 12 Agnes Ginges Centre for Molecular Cardiology at Centenary Institute, University of Sydney, Sydney, Australia; 13 University of Groningen Department of Genetics, University Medical Center Groningen, Groningen, The Netherlands; 14 Leon H. Charney Division of Cardiology, NYU School of Medicine, New York, NY US; 15 University of Colorado Anschutz Medical Campus, Aurora, CO US; 16 Cardiovascular Department, Azienda Sanitaria-Universitaria Giuliano Isontina (ASUGI), Trieste, Italy; 17 Department of Cardiology, University Heart Center, University Hospital, Zurich, Switzerland; 18 Department of Genetics, University Medical Center Utrecht, Utrecht, the Netherlands; 19 Netherlands Heart Institute, Utrecht, the Netherlands

## Abstract

**Purpose:**

The genetic architecture of Plakophilin 2 (*PKP2*) cardiomyopathy can inform our understanding of its variant pathogenicity and protein function.

**Methods:**

We assess the gene-wide and regional association of truncating and missense variants in *PKP2* with arrhythmogenic cardiomyopathy (ACM), and arrhythmogenic right ventricular cardiomyopathy (ARVC) specifically. A discovery data set compares genetic testing requisitions to gnomAD. Validation is performed in a rigorously phenotyped definite ARVC cohort and non-ACM individuals in the Geisinger MyCode cohort.

**Results:**

The etiologic fraction (EF) of ACM-related diagnoses from truncating variants in *PKP2* is significant (0.85 [0.80,0.88], *p* < 2 × 10^−16^), increases for ARVC specifically (EF = 0.96 [0.94,0.97], *p* < 2 × 10^−16^), and is highest in definite ARVC versus non-ACM individuals (EF = 1.00 [1.00,1.00], *p* < 2 × 10^−16^). Regions of missense variation enriched for ACM probands include known functional domains and the C-terminus, which was not previously known to contain a functional domain. No regional enrichment was identified for truncating variants.

**Conclusion:**

This multicohort evaluation of the genetic architecture of *PKP2* demonstrates the specificity of *PKP2* truncating variants for ARVC within the ACM disease spectrum. We identify the *PKP2* C-terminus as a potential functional domain and find that truncating variants likely cause disease irrespective of transcript position.

## INTRODUCTION

The yield of genetic testing in inherited cardiovascular disease relies on identification of disease-causative genes and the ability to distinguish pathogenic from benign variation therein. Efforts to define the genetic architecture of inherited cardiomyopathies have yielded significant improvement in our understanding of their most common genetic etiologies and population rarity.^1,2^ However, comprehensive position-specific variant pathogenicity assessment remains elusive. Recent efforts have characterized regional patterns in variant–disease association in genes including *TTN*, *MYH7*, *DSP*, and most recently *MYBPC3.*^3–7^ We showed that agnostic regional comparison of variants found in disease-associated cohorts versus the general population confirmed known functional domains and novel areas of interest in the gene *RBM20.*^8^ Here, we refine and apply this heuristic along with a gene-wide analysis to investigate the genetic architecture of a well-known cause of arrhythmogenic cardiomyopathy (ACM): Plakophilin 2 (*PKP2*).

*PKP2* is the most common gene associated with ACM, specifically its right dominant subform, arrhythmogenic right ventricular cardiomyopathy (ARVC).^9,10^ The PKP2 protein is part of the desmosome, critical for cell–cell adhesion, and has several known functional domains including the HR2 domain at its N-terminus as well as eight Armadillo repeats, thought to be involved in protein interactions.^11^ *PKP2* cardiomyopathy, like many genetic causes of cardiomyopathy, is inherited in an autosomal dominant fashion, and is thought to predominantly affect the right ventricle, therefore being specifically associated with ARVC.^5,12^ Prior observations in ARVC have suggested that loss of function *PKP2* variants explain a significant etiologic fraction (EF) of this disease.^1^

However, important questions regarding the genetic architecture of *PKP2* cardiomyopathy remain. First, although its major associated phenotype is ARVC, pathogenic and likely pathogenic (P/LP) variants in *PKP2* have also been associated with ventricular arrhythmia and sudden cardiac death without overt RV involvement.^13,14^ The EF of ACM explained by *PKP2* variants that is inclusive of these phenotypes remains to be investigated. Second, though P/LP *PKP2* missense variants have been reported and validated with functional studies, their overall impact in ACM and specifically ARVC at a population level is difficult to discern. A regional assessment for disease enrichment of *PKP2* missense and truncating variants may help to inform variant pathogenicity assessment.^1,15–17^

We investigated these questions in a multicohort study inclusive of a large database of clinical genetic testing, a rigorously phenotyped cohort of patients with definite ARVC, the population genomics database gnomAD,^18^ and the Geisinger MyCode cohort,^19^ which is a population cohort with available clinical phenotypes. We first delineate the EF of truncating and missense *PKP2* variants in individuals with ACM-associated diagnoses and ARVC specifically. We go on to examine the regional enrichment of ACM and ARVC probands with *PKP2* missense and truncating variants, respectively, compared to individuals with such variants in the general population. We illuminate the respective utility of clinical genetic testing data with and without rigorous clinical phenotyping for human genetics discovery, present a heuristic for regional interrogation of disease-associated variation, and identify a potential novel functional domain of *PKP2*.

## MATERIALS AND METHODS

### Discovery data set

#### Clinical genetic testing cohorts

**Fig. 1:**
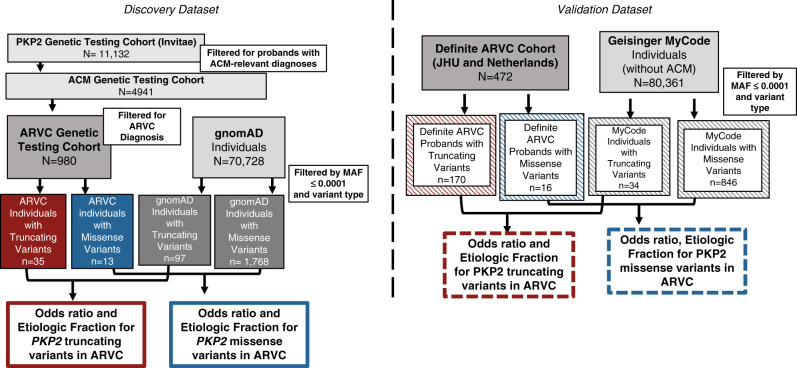
Study design for determination of etiologic fraction (EF). Two independent data sets were used, one for discovery and one for validation. In the discovery data set, individuals undergoing genetic testing at Invitae, Inc. were filtered for probands carrying an arrhythmogenic cardiomyopathy (ACM)–associated diagnosis and further filtered for those carrying an arrhythmogenic right ventricular cardiomyopathy (ARVC) diagnosis specifically. Of these, EF was determined for those carrying truncating and missense variants as compared to members of the general population carrying rare *PKP2* variants (minor allele frequency [MAF] ≤ 0.0001). In the validation data set, patients with a definite diagnosis of ARVC (Johns Hopkins [JHU] and Netherlands cohorts) were included and those with a pathogenic or likely pathogenic variant in *PKP2* were compared to individuals in the Geisinger MyCode cohort with *PKP2* variants but without ACM-related diagnoses.

We mined free text and ICD-10 codes from 11,132 individual de-identified genetic tests from Invitae, Inc. for probands with ACM-associated diagnoses who were sequenced for *PKP2*. As there is no universally accepted disease definition of ACM, we defined ACM probands as those carrying a diagnosis of cardiomyopathy, ventricular arrhythmia, or sudden cardiac death (Supplementary Table 1). We identified 4,941 probands carrying ACM-associated diagnoses (“ACM Genetic Testing Cohort,” Fig. 1). Of these, 980 carried a specific diagnosis of ARVC (“ARVC Genetic Testing Cohort,” Fig. 1), though it was not possible to determine from available data whether these probands met 2010 Task Force Diagnostic Criteria.^20^ In this discovery data set, all detected variants were included, regardless of pathogenicity adjudication, to allow for agnostic evaluation of disease association. Stop-gain and frameshift variants were included in the truncating variant group, and only nonsynonymous missense variants were included. Splice site variants were not included given unknown transcript consequences of most variants in predicted splice sites. Only probands carrying variants sufficiently rare to cause disease were included (minor allele frequency [MAF] ≤ 3.6 × 10^−5^). This cutoff is based on the population rarity of the most common pathogenic variant in *PKP2* (c.2146-1G>C) in gnomAD, and a published allele frequency calculator that integrates inheritance, allelic and genetic heterogeneity, and penetrance.^2^

**Fig. 2:**
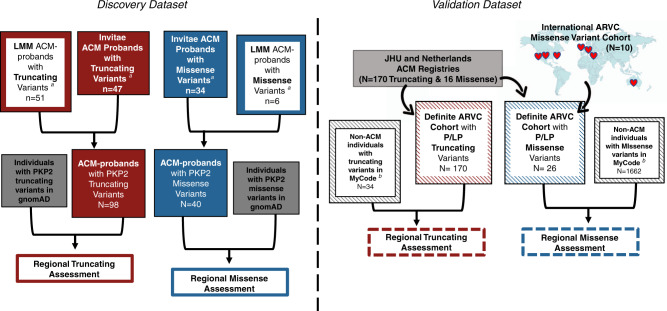
Study design for regional assessment of missense and truncating variant distribution. The discovery data set here was supplemented with genetic tests in arrhythmogenic cardiomyopathy (ACM) probands from the Laboratory for Molecular Medicine (LMM). Invitae probands and definite ARVC cohorts described in Fig. 1. The validation data set was supplemented with the addition of the International ARVC Missense Variant Cohort (*N* = 10 pathogenic/likely pathogenic [P/LP] *PKP2* missense variants). ^a^Minor allele frequency (MAF) ≤ 3.6 × 10^−5^. ^b^MAF ≤ 0.001. ARVC arrhythmogenic right ventricular cardiomyopathy, JHU Johns Hopkins cohort.

To increase statistical power for regional analysis of missense and truncating variants, we supplemented the ACM Genetic Testing Cohort with additional ACM probands with variants of uncertain significance (VUS) and P/LP variants in *PKP2* from the Laboratory for Molecular Medicine (LMM, Fig. 2). We cannot completely exclude the possibility that the de-identified probands from these two testing providers were related. However, only two probands sharing the same variant from these respective providers were included (c. 2386T>C [p.C796R]). Resultant pools of individuals were formed for ACM-associated truncating variant probands (*N* = 98, solid red box, Fig. 2) and ACM-associated missense variant probands (*N* = 40, solid blue box Fig. 2). LMM genetic tests were not included in EF assessment because the total number of ACM probands tested at LMM was not obtained.

#### Genome Aggregation Database (gnomAD) Population Genomics Cohort

Individuals carrying nonsynonymous missense and truncating variants in *PKP2* from 123,136 exomes and 15,496 genomes in the Genome Aggregation Database (gnomAD V2.1.1)^21^ were collated. On average, sequencing from 70,728 individuals passed quality control at each coding position of the *PKP2* transcript, and we defined this as the size of the general population sequenced. For purposes of calculating the EF of truncating and missense variants for ARVC and ACM, only individuals carrying variants with gnomAD MAF ≤ 0.0001 were included to avoid counting compound heterozygotes or variants in *cis* as more than one individual. One thousand seven hundred sixty-eight individuals carrying *PKP2* missense and 97 carrying *PKP2* truncating variants were identified in gnomAD (light gray boxes, Fig. 1). For regional analyses, differential MAF restrictions were used to optimize the heuristic (see Supplementary Methods).

### Validation data set

To complement the discovery data set described above, we compiled rigorously phenotyped data sets for validation analyses. These provide more precise estimation of the odds of ARVC association with specific variant classes and locations based both on inclusion of only definite ARVC in the disease cohort as well as excluding ACM-relevant phenotypes from the control cohort (Geisinger MyCode).

#### Definite ARVC Cohort

We assembled a large group of probands with definite ARVC.^20^ This cohort is comprised of two contributing groups:

##### Johns Hopkins University (JHU)/Netherlands (NL) ACM Registries

The Johns Hopkins (JHU) ARVC Registry (arvd.com) and Netherlands ACM Registry (acmregistry.nl) both prospectively enroll individuals with ACM, most typically ARVC, as well as individuals who are at risk for these conditions based on genotype and clinical features.^22^ *PKP2*-variant-carrying probands from the JHU/NL registries were drawn from a recent study that characterized P/LP desmosomal variants among patients who met definite 2010 Task Force Criteria for ARVC.^20,23^ For this study, we retained only P/LP nonsense and frameshift variants in *PKP2*; splice variants and large deletions were excluded.

##### International ARVC Missense Variant Cohort

Given the rarity of P/LP missense variants in *PKP2*, in addition to probands with missense variants from the NL and JHU Registries we also included data from multiple international ARVC centers of excellence in Europe, the United States, and Australia (Zurich ARVC Program, Arrhythmogenic Cardiomyopathy program at the University College London Institute of Cardiovascular Science, Stanford Center for Inherited Cardiovascular Disease, The Australian Genetic Heart Disease Registry, The Familial Cardiomyopathy Registry [University of Colorado–University of Trieste, Italy]) to assemble a cohort of probands with definite ARVC per 2010 Task Force Criteria with similarly rare *PKP2* missense variants (MAF ≤ 3.6 × 10^−5^) (Supplementary File 2).^20^ Probands with isolated left ventricular involvement (i.e., arrhythmogenic left ventricular cardiomyopathy, ALVC) that did not meet 2010 Task Force Criteria were not included. These data were used exclusively for the regional assessment of missense variation.

In total, the Definite ARVC Cohort included 170 *PKP2* variant–carrying probands with rare nonsense or frameshifting insertions/deletions from the recent study by van Lint et al.^23^ (Figs. 1 and 2, red hatched boxes). Sixteen rare missense variant–carrying probands also from van Lint et al.^23^ were included in the EF validation analysis (Fig. 1, blue hatched box) and 10 additional definite ARVC probands harboring missense variants from international centers were added for the regional missense variant analysis (total *N* = 26, Fig. 2, blue hatched box). Summary clinical characteristics are provided in Supplementary Table 2.

#### Geisinger MyCode Cohort

The MyCode Community Health Initiative is a precision health project of Geisinger Health System of Pennsylvania.^19^ Consenting participants provide biospecimens for broad research use, including linkage of results to their Geisinger electronic health record (EHR) (Epic). Exome sequence data have been completed for approximately 145,000 participants through the DiscovEHR collaboration with Regeneron Genetics Center, as described elsewhere.^24,25^ MyCode therefore represents a sequenced population with comprehensive clinical phenotyping. We reviewed the project-level variant call file (VCF) for 132,890 participants with at least 1 year of follow-up in the EHR to identify all rare missense, frameshift, and nonsense variants in *PKP2*, based on annotations from Ensembl VEP. We excluded all individuals with closer than 3rd degree relatedness based on the exclusive focus on probands within the clinical cohorts. Finally, to improve specificity in this control population, patients with diagnoses of “other/unspecified” cardiomyopathy, ventricular arrhythmia/tachycardia/fibrillation, bundle branch block, or cardiac arrest were excluded (*N* = 4721, Figs. 1 and 2; See Supplementary Table 1 for specific ICD-10 codes), leaving 80,361 individuals in the final analyses.

### Regional assessment of variants for disease association

We designed a sliding window heuristic, building on methods we have described previously.^8^ Using the number of probands and individuals identified in the Discovery data set, we estimated the statistical power provided for a given sliding window size in base pairs (bp) assuming uniform distribution of variants across the coding transcript. Window size was determined based on the following parameters: desired power 0.80, alpha error 0.01, detectable odds ratio 4.5 using the calculator available at http://openepi.com/Power/PowerCC.htm (Supplementary Table 3). We then compared the fraction of individuals within each of these windows (number of individuals with variants falling in a window/number of total individuals in respective database) between the ACM genetic testing cohort and gnomAD individuals, calculating an odds ratio for enrichment of ACM probands versus gnomAD individuals (and again for Definite ARVC probands versus MyCode participants). Sliding windows were then shifted by a single base pair down the length of the coding transcript to increase the granularity of identification of regional enrichment. More detail regarding the progressive development of the heuristic is included in the Supplementary Methods, Supplementary Fig. 1, and Supplementary Table 4.

### Statistical analyses

For comparison of proportions with *n* < 100, a Fisher exact test was performed, otherwise a Chi-squared test was used. EF was calculated as a function of the odds ratio (OR): EF = (OR – 1/OR). Correction for multiple testing was performed using a Benjamini–Hochberg method with false discovery rate (FDR) of 0.01 (or 0.05 for validation). All analyses were performed using Stata 14 or R. Bespoke code written for these analyses is available upon request.

## RESULTS

### *PKP2* truncating variants explain a significant etiologic fraction of ACM, most specifically ARVC

The OR for association of each *PKP2* variant type (truncating or missense) with an ARVC diagnosis in the Invitae cohort was calculated (*N* = 980, Fig. 1). Truncating variants were highly enriched in probands with an ARVC diagnosis as compared to gnomAD (Table 1). Rare *PKP2* missense variants were less likely to be identified in the ARVC genetic testing cohort than in gnomAD. Next, this result was assessed in the validation database (JHU/Netherlands ARVC Registries and MyCode), addressing the hypothesis that high fidelity clinical phenotyping of these cases and elimination of affected individuals in the general population would more precisely measure the effect. As suspected, this yielded a significantly higher odds of disease association and EF (OR 853 [581,1,251]; EF 1.00 [1.00,1.00]; *p* < 2 × 10^−16^, Table 1). Missense variants in *PKP2* were also enriched in this validation data set, though with a much smaller effect size (OR 3.2 [1.8,5.3]; EF 0.69 [0.44,0.81]; *p* = 9.1 × 10^−5^). Of note, these may yet be slight underestimates of the actual EF of *PKP2* variants in ACM, as only ICD-10 codes found in disease cohorts were excluded (Supplementary Table 1), and therefore the MyCode population may still include some individuals with more generalized cardiac diagnoses.

**Table 1:** Truncating variants in *PKP2* are more strongly associated with ACM phenotypes than missense variants.

Disease group^a^	*n* (%)	gnomAD^b^ *N* = 70,728 *n* (%)	OR (95% CI)	EF (95% CI)	*p* value
**Genetic testing ACM (*N*** = **4941)**					
Truncating	47 (0.9%)	98 (0.1%)	6.9 [4.9,9.8]	0.85 [0.8,0.9]	<2 × 10^−16^
Missense	137 (2.8%)	1768 (2.2%)	1.1 [0,9,1.3]	0.09 [0,0.2]	0.23
**Genetic testing ARVC (*N*** = **980)**					
Truncating	35 (3.5%)	98 (0.1%)	26.7 [18.1,39.5]	0.96 [0.94,0.97]	<2 × 10^−16^
Missense	13 (1.3%)	1768 (2.2%)	0.5 [0.3,0.9]		0.02
**Definite ARVC (*N*** = **472)**	***n*(%)**	**MyCode**^**b**^***N*** = **80,361** ***n*** **(%)**	**OR (95% CI)**	**EF (95% CI)**	***p*value**
Truncating	170 (36%)	34 (0.04%)	853 [581,1,251]	1.00 [1.00,1.00]	<2 × 10^−16^
Missense	16 (3.4%)	846 (1.1%)	3.19 [1.8,5.3]	0.69 [0.44,0.81]	9.1 × 10^−5^

*ACM* arrhythmogenic cardiomyopathy, *ARVC* arrhythmogenic right ventricular cardiomyopathy, *CI* confidence interval *EFetiologic* fraction, *OR* odds ratio.

^a^Probands, ACM-associated or ARVC specific diagnosis by ICD-10 or requisition (Invitae, Inc.), MAF ≤ 0.0001.

^b^Minor allele frequency (MAF) ≤ 0.0001, Relatives closer than 3rd degree and individuals with ACM-relevant diagnoses removed from MyCode.

To investigate whether the inclusion of probands with any ACM-associated phenotype (not only ARVC) was adequate to detect an effect of *PKP2* truncating variants in ACM, we performed the same analysis using data from the ACM genetic testing cohort (Supplementary Table 1 inclusive of, but not exclusive to ARVC; Fig. 1, *N* = 4,941). Of these probands, 47 had truncating variants in *PKP2*, yielding a modest enrichment for disease association of truncating variants compared to gnomAD (OR 6.9 [4.9,9.8]; EF 0.85 [0.80,0.88]; *p* < 0.0001). The large decrease in effect size for ACM versus ARVC specifically highlights the specificity of *PKP2* truncating variants for an ARVC phenotype, a finding that has been reported in smaller cohorts.^12^

### Regions of PKP2 enriched for ACM-associated missense variation include known functional domains and the previously unrecognized C-terminus

**Fig. 3:**
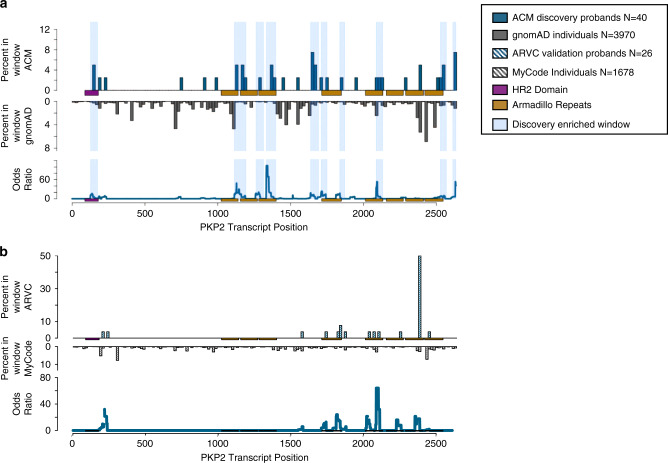
Regional assessment of missense variants in *PKP2* identifies potential hotspots for pathogenic variation. (**a**) Arrhythmogenic cardiomyopathy (ACM) genetic testing cohort (blue, *N* = 40, minor allele frequency [MAF] ≤ 3.6 × 10^−5^) vs. gnomAD (gray, *N* = 3970, MAF ≤ 0.001) Odds of inclusion of disease associated variant. (**b**) International ARVC Missense Variant Cohort (striped blue, MAF ≤ 3.6 × 10^−5^, *N* = 26) vs. Geisinger MyCode cohort (striped gray, *N* = 1678, MAF ≤ 0.001). Light blue shading indicates regional disease enrichment, false discover rate (FDR) = 0.01. ARVC arrhythmogenic right ventricular cardiomyopathy.

We went on to examine potential regional enrichment for disease associated missense and truncating variation in *PKP2*. Using progressively restrictive inclusion of population variants and sliding window size based on statistical power calculations, we developed a heuristic for regional evaluation of disease proband enrichment compared to population control cohorts (additional detail in Supplementary Methods). Initial examination of regional distribution of missense variants in the discovery data set identified several disease-enriched windows along the *PKP2* transcript (Fig. 3a). These capture not only most Armadillo repeats, but also the previously reported HR2 domain. Additionally, the *PKP2* C-terminus was also highly enriched across different conditions of sliding window size and MAF restriction (Supplementary Figure 1).

Given the specificity of *PKP2* cardiomyopathy for the ARVC phenotype in particular, we hypothesized that of the ACM probands and *PKP2* missense variants in the Clinical Genetic Testing Cohort (*N* = 40, Fig. 2, solid blue box), those with ARVC-specific diagnosis would be enriched in predicted windows (*N* = 16). We found that ARVC probands were marginally enriched in predicted windows over those with other ACM-related diagnoses (OR 5 [0.8,53], *p* = 0.048). Interestingly, this did not hold true when regions identified with lenient or strict gnomAD MAF restrictions were examined (Supplementary Figure 1, MAF ≤ 0.01:OR 1.4 [0.17,17.4], *p* = 0.7; MAF ≤ 3.6 × 10^−5^: OR 1.9 [0.41,8.6], *p* = 0.34).

### Regions of PKP2 enriched for ACM-associated missense variation in the discovery cohort are also enriched for pathogenic and likely pathogenic variant ARVC patients

To validate these findings further, we performed a regional analysis using the same window size and MAF restrictions in the validation data set (Fig. 3b). A well-described pathogenic founder missense variant, c.2386T>C (p.C796R), was overrepresented in these definite ARVC probands. For this reason, only this region of the transcript met statistical significance at FDR 0.01. However, the odds of disease association in this analysis do reproduce several armadillo repeats and also display a peak near the HR2 domain, overlapping with many of the enriched windows identified in the discovery data set analysis.

Of note, the significant peak around the c.2386T>C variant observed in the validation cohort was not observed in the discovery cohort. We further investigated this finding in the discovery cohort, and found that the regions within 33 bp of this variant were enriched for variation in gnomAD. We hypothesized that this was largely driven by a variant prevalent specifically in participants of African and Latino ancestry (MAF 7 × 10^−4^, p.Thr798Ala). Given that the c.2386T>C founder variant, and indeed much of ARVC is described in individuals of non-Finnish European ancestry, we then performed this regional analysis including only individuals of non-Finnish European ancestry from gnomAD (MyCode participants are >90% European ancestry). Despite representation of the founder variant in the ACM genetic testing cohort, this did not significantly change our findings, and specifically did not identify the region around the founder variant as enriched for disease (likely due to 11 non-Finnish European individuals carrying c.2392A>G [p.Thr798Ala], Supplementary Figure 2). As the MyCode registry includes 93.5% individuals of European ancestry, we did not repeat an ancestry-specific analysis in the validation data set (the ancestry admixture of each cohort is included in Supplementary Table 5).

### Assessment of truncating variation identifies no regional disease enrichment

Regional association of truncating variation has previously illuminated functional motifs (e.g., internal promoters) in other genes.^3^ Therefore, we examined the distribution of variants across the transcript and found no linear association of transcript location with enrichment for ACM versus population associated truncating variation (*R*^2^ = 0.003, *p* = 0.42).

**Fig. 4:**
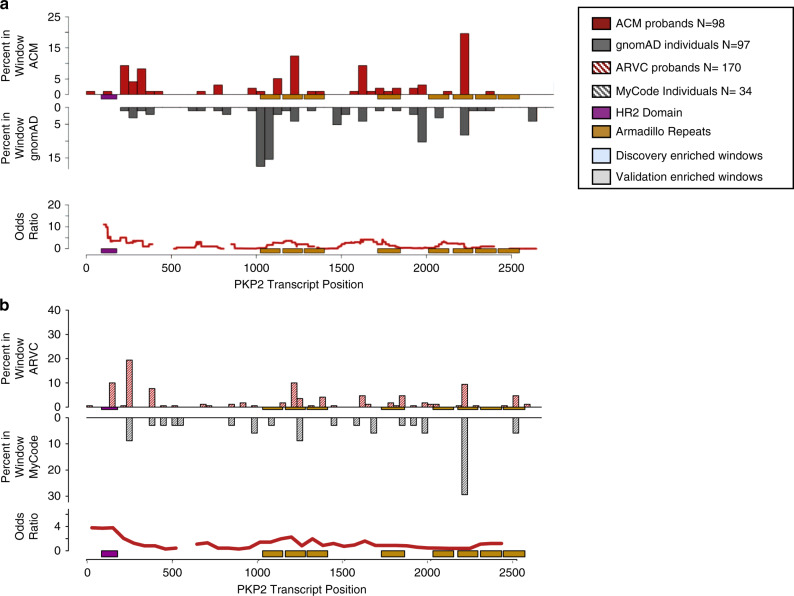
Regional assessment of truncating variants in PKP2 shows no association of transcript location with arrhythmogenic cardiomyopathy (ACM) phenotypes. (**a**) Clinical genetic testing cohort (red, *N* = 98) vs gnomAD (gray, *N* = 97) Odds of inclusion of disease associated variant. (No statistically significant windows at false discovery rate [FDR] 0.01. *R*^2^ = 0.003, *p* = 0.42.) (**b**) Johns Hopkins (JHU)/Netherlands cohort (hatched red) vs. Geisinger cohort (hatched gray) (no statistically significant windows at FDR 0.01). ARVC arrhythmogenic right ventricular cardiomyopathy.

We went on to perform a regional analysis for enrichment of ACM-associated truncation. Due to the very restricted sample size in gnomAD (*N* = 97), we used a 132-bp window for this analysis. We did not find any windows enriched for ACM-associated variation, consistent with the hypothesis that truncating variation is likely disease associated regardless of its location (Fig. 4a, b). Because there was a notable increase in the OR of windows around the HR2 domain, we repeated this analysis with 33-bp windows to ensure enough precision with the sliding window size, and still found no significant enrichment. We did note a significant overrepresentation of truncating variation in the general population at positions c.926-c.1004, driven by 12 individuals each with p.Ala325TrpfsTer28 and p.Gln323ArgfsTer12 respectively. Based on publicly available data in gnomAD, it is not possible to determine whether these variants exist in independent individuals or are carried in a common haplotype. We examined these variants in MyCode and found that they were part of a common haplotype in 41 individuals that leads to the resolution of predicted truncation, instead causing two missense variants in tandem: p.Gln323_Ala324delinsArgLeu. These individuals in the MyCode cohort were recoded as having missense variants for all analyses.

## DISCUSSION

Here, we employ clinical genetic testing cohorts, highly phenotyped definite ARVC cohorts, and population genomic data with and without associated clinical information to describe the genetic architecture of *PKP2* cardiomyopathy. We show that truncating variation at any position along the *PKP2* transcript explains a large EF of *PKP2* cardiomyopathy, and highlight missense variation at the C-terminus of the *PKP2* transcript encoding a potential unrecognized functional domain. We find that the magnitude of variant disease contribution is most accurately measured with highly specific disease phenotypes and that elimination of disease-associated individuals from control populations increases this specificity. Lastly, we develop a balanced heuristic for the regional comparison of human disease and population-associated genetics to illuminate potential functional protein domains associated with disease. These findings have direct clinical implications. Regional mapping of novel *PKP2* variants to areas of the gene enriched for disease association in this study may aid in variant interpretation. In combination with this, the knowledge that truncating variants are very likely disease causing and particularly for ARVC will aid in the diagnosis and care of patients with *PKP2* cardiomyopathy and their families.

### *PKP2* truncating variants explain a large EF of ARVC, and are not regionally clustered

We show, in two independent data sets, that *PKP2* truncating variants explain a large EF of ARVC, and that there is no relationship between their transcript position and their likelihood of disease association. These findings reinforce prior evidence that *PKP2* truncating variation explains the largest EF of ARVC in an independent, highly phenotyped population.^1^ Our findings also demonstrate that with exclusion of patients carrying disease-relevant phenotypes from the control population in MyCode and high-fidelity clinical phenotyping for definite ARVC, the true EF of *PKP2* truncating variants is higher than predicted by our less well phenotyped clinical genetic testing cohort. However, these findings also indicate that discovery can *detect* these effects using larger but less clinically defined databases, though the effect size of such associations is likely to be underestimated. To date, our knowledge of the spectrum of disease caused by the variants we identify has been limited by our approach, which tests only those patients fitting a specific and often rigidly defined clinical phenotype. As we move into an era of genome-first approaches to diagnosis, we may find that where the specificity of a given genotype for a particular clinical phenotype is high, its population penetrance remains small.^26^ It is possible that other relevant phenotypes exist in patients carrying these pathogenic alleles (e.g., sudden cardiac arrest in the absence of diagnostic ARVC imaging criteria), and contribute to that gap in penetrance of the expected clinical phenotype. Inclusion of broader disease definitions in our study of rare disease may be useful to illuminate some of these gaps.

### Human genetics illuminates known and potential functional domains in PKP2

Our regional analysis identifies potential hotspots for missense variation in most of the Armadillo repeats of *PKP2*, helical structures that were first described in the β-catenin crystal structure and are thought to facilitate protein–protein interactions with acidic substrates.^27^ We again implicate the HR2 domain, critical for desmosomal assembly.^11^ That this regional analysis did not identify the region surrounding the functionally validated *PKP2* founder variant c.2386T>C; p.C796R^28^ in the discovery cohort, but was able to detect it in the validation cohort, requires consideration. This may indicate that the p.C796 position is itself critical to protein structure (instability of PKP2 p.C796R has been reported^28^) but that it does not otherwise lie in a functional domain encompassing more than this residue. It may also indicate that exclusion of relevant diagnoses in the population made possible in the MyCode cohort is necessary for detection of this regional pathogenicity. The overrepresentation of this variant in the definite ARVC cohort due to founder effect also likely drives this finding, as the ACM genetic testing cohort has a more diverse ancestry admixture (Supplementary Fig. 2, legend). Finally, the exclusion of non-ARVC phenotypes from the validation cohort may result in more ARVC-specific regional enrichment, whereas the discovery cohort may include variants that might be more associated with early arrhythmia or ALVC phenotypes.

In addition, our analysis illuminates the C-terminus as a potential functional domain. This implies that both the N- and C-termini of the *PKP2* protein are functionally critical. That disease-associated truncating variants are not enriched nearer the N-terminus, but rather distributed throughout may also support this conclusion. Although C-terminus enrichment was not recapitulated in the validation data set, this is to be expected given the restriction of the validation cohort to P/LP variants, and the previously unrecognized significance of this region. At present, molecular investigation of the *PKP2* C-terminus has been limited, and specific knowledge of its protein domains, especially in the cardiomyocyte, is lacking. In the PKP2 homologue Plakophilin 1, the C-terminus is necessary for desmosome assembly in epithelial cells.^29^ Therefore, missense variants in this region of *PKP2* may be more functionally consequential in ACM than previously thought. Given its centrality in the desmosome, interactors of *PKP2* may require the C-terminus. Further investigation of these molecular interactions will illuminate not only our understanding of disease mechanisms, but also will serve to reinforce missense variant interpretation.

### Limitations

As with any study in rare disease, our findings are limited by the small sample size available to study granular hypotheses. We have described above the measures taken to avoid selection bias and other bias associated with studying rare disease. Though we have performed sensitivity analyses to ensure that our novel findings are not related to differential ancestry mix between cases and controls, the effect of ancestry on variant effect remains difficult to study in existing biobanks and patient populations with limited admixture. Therefore our findings may be less applicable in patients of non-European ancestry. It is important to note that while this analysis offers a regional assessment of variant-to-disease association and therefore highlights the power of human genetics to illuminate protein structure and function, it may supplement but not replace significant requisite data in the adjudication of individual variant pathogenicity as outlined by the American College of Medical Genetics and Genomics.^30^

## Conclusion

We use human genetics to illuminate detailed gene-wide and region-specific variant–disease association in *PKP2* cardiomyopathy. As we move toward a genome-first era of medicine, the development and broad application of the methods presented here can provide a platform for the incorporation of human genetics into the position-specific evaluation of variant pathogenicity at scale. As such, the analysis presented here seeks to improve diagnosis and understanding of disease mechanisms by linking genetic discovery directly to clinical observations.

## Supplementary Information

Supplementary Information

Supplementary File 1 _variant list

Supplementary File 2_ International ARVC Missense Cohort

Supplementary File 3_ Regional ORs

## Data Availability

All data have been made available in de-identified form in the included supplementary files. Any additional queries can be directed to the corresponding authors.
