# Correction to: The genetic architecture of Plakophilin 2 cardiomyopathy

**DOI:** 10.1038/s41436-021-01298-4

**Published:** 2021-08-18

**Authors:** Annika M. Dries, Anna Kirillova, Chloe M. Reuter, John Garcia, Hana Zouk, Megan Hawley, Brittney Murray, Crystal Tichnell, Kalliopi Pilichou, Alexandros Protonotarios, Argelia Medeiros-Domingo, Melissa A. Kelly, Aris Baras, Jodie Ingles, Christopher Semsarian, Barbara Bauce, Rudy Celeghin, Cristina Basso, Jan D. H. Jongbloed, Robert L. Nussbaum, Birgit Funke, Marina Cerrone, Luisa Mestroni, Matthew R. G. Taylor, Gianfranco Sinagra, Marco Merlo, Ardan M. Saguner, Perry M. Elliott, Petros Syrris, J. Peter van Tintelen, Regeneron Genetics Center, Cynthia A. James, Christopher M. Haggerty, Victoria N. Parikh

**Affiliations:** 1 Stanford Center for Inherited Cardiovascular Disease, Division of Cardiovascular Medicine, Department of Medicine, Stanford University School of Medicine, Stanford, CA USA; 2 Invitae, Inc, San Francisco, CA USA; 3 Laboratory for Molecular Medicine, Mass General Brigham Personalized Medicine, Cambridge, MA USA; 4 Dept. Pathology, Massachusetts General Hospital, Harvard Medical School, Boston, MA USA; 5 Division of Cardiology, Department of Medicine, Johns Hopkins University, Baltimore, MD USA; 6 Dept. of Cardiac-Thoracic-Vascular Sciences and Public Health, University of Padua, Padua, Italy; 7 Centre for Heart Muscle Disease, Institute of Cardiovascular Science, University College London, London, UK; 8 SwissDNALysis-Cardiogenetics, Dübendorf Switzerland, Zurich, Switzerland; 9 Geisinger, Danville, PA USA; 10 Regeneron Genetics Center, Tarrytown, NY USA; 11 Cardio Genomics Program at Centenary Institute, The University of Sydney, Sydney, Australia; 12 Agnes Ginges Centre for Molecular Cardiology at Centenary Institute, University of Sydney, Sydney, Australia; 13 University of Groningen Department of Genetics, University Medical Center Groningen, Groningen, The Netherlands; 14 Leon H. Charney Division of Cardiology, NYU School of Medicine, New York, NY US; 15 University of Colorado Anschutz Medical Campus, Aurora, CO US; 16 Cardiovascular Department, Azienda Sanitaria-Universitaria Giuliano Isontina (ASUGI), Trieste, Italy; 17 Department of Cardiology, University Heart Center, University Hospital, Zurich, Switzerland; 18 Department of Genetics, University Medical Center Utrecht, Utrecht, the Netherlands; 19 Netherlands Heart Institute, Utrecht, the Netherlands

Correction to: *Genetics in Medicine* 2021; 10.1038/s41436-021-01233-7 published online 12 June 2021

Due to a processing error Cynthia James, Brittney Murray, and Crystal Tichnell were assigned to the wrong affiliation.

Cynthia James, Brittney Murray, and Crystal Tichnell have as their affiliation 5 Division of Cardiology, Department of Medicine, Johns Hopkins University, Baltimore, MD, USA.

In addition Hana Zouk, Megan Hawley, and Birgit Funke were assigned only to affiliation 3; they also have affiliation 4 Department of Pathology, Massachusetts General Hospital, Harvard Medical School, Boston, MA, USA.

The original article has been corrected.

