# Supplementary material for: The genetic architecture of Plakophilin 2 cardiomyopathy
Source: Genet Med. 2021 Jun 12;23(10):1961–8. doi: 10.1038/s41436-021-01233-7 (PMC8486657; doi:10.1038/s41436-021-01233-7)
Supplement: Supplementary file 1 — Supplementary Information [file 41436_2021_1233_MOESM1_ESM.docx]

#

#

#

#

#

#

#

#

# Supplementary Information

# The Genetic Architecture of Plakophilin 2 Cardiomyopathy

Annika M. Dries^1^, Anna Kirillova^1^, Chloe M. Reuter MS^1^, John Garcia PhD^2^, Hana Zouk PhD^3^, Megan Hawley^3^, Brittney Murray MS CGC^4^, Crystal Tichnell MGC RN^4^, Kalliopi Pilichou PhD^5^, Alexandros Protonotarios MD^6^, Argelia Medeiros-Domingo^7^, Melissa A. Kelly MS CGC^8^, Aris Baras MD^9^, Jodie Ingles PhD MPH^10^, Christopher Semsarian MBBS PhD MPH^11^, Barbara Bauce, MD PhD^5^, Rudy Celeghin PhD^5^, Cristina Basso MD PhD^5^,Jan DH Jongbloed PhD^12^, Robert L. Nussbaum MD^2^, Birgit Funke PhD^3^, Marina Cerrone MD^13^, Luisa Mestroni MD^14^, Matthew RG Taylor, MD PhD^14^, Gianfranco Sinagra MD^15^, Marco Merlo MD^15^, Ardan M. Saguner MD^16^, Perry M. Elliott MD FRCP^6^, Petros Syrris PhD^6^, J. Peter van Tintelen MD PhD ^17,18^, Regeneron Genetics Center, Cynthia A. James PhD CGC^4^*, Christopher M. Haggerty, PhD^8^*, and Victoria N. Parikh MD^1^*

#

#

#

#

#

#

#

#

# Supplementary Methods

***Rational development of a regional pathogenicity assessment heuristic for missense variants***

Evaluation of regional enrichment of disease-associated variation for this purpose poses a challenge due to multiple competing interests including: 1. identifying small, potentially functional regions of the transcript with precision, 2. maintaining enough statistical power with small cohorts to identify expected effect sizes, 3. eliminating variants from comparison populations that are common enough to co-exist with other variants in the same individual while, 4. at the same time maintaining enough common variants to indicate areas of the transcript tolerant to variation. These interests compete with each other with respect to sliding window size (1 and 2 above) and restriction of variant inclusion from the general population by MAF (3 and 4). We therefore iterated across these two variables using a sliding window design as we have previously described.[^8^](https://paperpile.com/c/Mpu3bZ/JNSI) Notably, this prior work evaluated enrichment for observed variants. Here, we investigate enrichment of disease probands and population individuals carrying these variants.

We began by identifying a window size with adequate statistical power to detect the odds ratios previously observed in our variant level analysis (4.5, see Supplementary Table 3). All probands included had variants with population MAF≤3.6x10^-5^ as described in the main text. However, as is described in the preceding paragraph, the effect of inclusion of individuals from gnomad based on variant rarity on our findings required investigation. We first determined the statistical power for each window size when including individuals from gnomad with variants of variable MAF. In total, individuals in gnomAD carrying missense variants at each level of restriction included MAF≤ 0.01 N=12,408; MAF ≤ 0.001 N=3970; MAF ≤ 3.6x10^-5^ N=1020; solid dark grey boxes, Figure 2). All truncating variants in gnomAD occurred at MAF ≤ 3.6x10^-5.^

We determined that for lenient (MAF≤0.01), intermediate (MAF≤0.001) and strict (MAF≤3.6x10^-5^) population MAF restriction, a sliding window size of 132 bp would provide a power of 88-90% to detect this odds ratio, assuming equal distribution of variants across the transcript (Supplementary Table 3). Using this 132 bp sliding window and varying the gnomAD MAF restriction, we identified regions representing 50%, 25% and 1.4% of the transcript that were enriched for ACM-associated variation respectively (Supplementary Figure 1 and Supplementary Table 4). The etiologic fraction of ACM in individuals with *PKP2* variant in these regions fell precipitously with MAF restriction, as did the association of these regions with known functional domains. However, the odds ratios detected by this method were much higher than previously observed with a variant-level regional analysis, [^8^](https://paperpile.com/c/Mpu3bZ/JNSI) and we therefore sought to increase the precision of our method by decreasing the sliding window size.

We found based on the same assumptions that we had adequate power to detect an OR of 20 with a sliding window size of 33 bp regardless of gnomAD MAF (Supplementary Table 3). We therefore repeated the regional analysis with this smaller window size at each MAF restriction described for individuals from gnomad. Using this method, we were able to capture not only more of the known functional domains of PKP2 within ACM-enriched windows, but were also able to preserve a more acceptable etiologic fraction (Supplementary Figure 1 and Supplementary Table 4).

Overall, we evaluated the specificity of each combination of window size (132bp vs 33 bp) and gnomAD MAF restriction for the capture of known functional domains and enrichment of ACM-associated patients in highly predicted windows (Supplementary Table 4). We conclude that a smaller window size offers increased precision in capturing known functional domains and explains a larger etiologic fraction of ACM in individuals with *PKP2* variants without sacrificing the statistical power necessary to detect the odds ratios measured. Using these smaller windows, we also show that restricting included individuals from the population comparison group to those with rare variation allows more precise identification of ACM-enriched regions. To strike a balance between maintaining the etiologic fraction captured and precisely identifying functional domains, we decided to validate our findings from the 33 bp windows with the MAF of gnomad variants restricted to those with MAF≤0.001 (Figure 3).

#

#

# Supplementary Tables and Figures

| **ACM phenotype variable criteria** | **ICD-10 codes:** |
| --- | --- |
| ARVC | I42.8 - other cardiomyopathy (ARVC) |
| DCM | I42.0 – DCM |
| Cardiomyopathy | I42.9 - Cardiomyopathy, unspecified |
| VT | I47.2 - VT |
| Sudden Cardiac Arrest/Death | I46.9 - Sudden Cardiac Arrest |
| VF | I49.01 - VF |

**Supplementary Table 1.** ACM-related Diagnoses and ICD-10 codes used for inclusion in the ACM genetic testing cohort (discovery dataset) and to exclude affected individuals in the Geisinger MyCode cohort (validation dataset).

| **Clinical Characteristics** | **International ARVC Missense Cohort**  **Mean±SD or Cases(%)** | **JHU/Netherlands ARVC Registry^23^**  **Mean±SD or Cases(%)** |
| --- | --- | --- |
| Sex  Male | 16 (62%) | 209(64.9) |
| Age at Presentation  (years) | 33.7±16.3 | 34±15 |
| Definite Diagnosis by ARVC Task Force Criteria | 26 (100%) | 322 (100%) |
| Repolarization  Major  Minor  None | 18 (69%)  3 (12%)  5 (19%) | 243 (82%)  16 (5.4%) |
| Depolarization  Major  Minor  None | 1 (4%)  15 (58%)  10 (38%) | 31 (11%)  129 (45.9%) |
| Arrhythmia  Major  Minor  None  Not Collected | 11 (42%)  9 (35%)  5 (19%)  1 (4%) | 121 (41.6%)  127 (43.6%) |
| Imaging/Structure  Major  Minor  None | 14 (54%)  4 (15%)  8 (31%) | 185(60.5%)  36 (11.8%) |
| Family History  Major  Minor  None | 6 (23%)  1 (4%)  19 (73%) | 322(100%) |
| Sustained VA (14-65 yrs) | 17 (65%) | 189 (59.2%) |
| Heart Transplant or Death | 3 (12%) | 33 (10.4%) |

**Supplementary Table 2. Summary of clinical characteristics of Definite ARVC Cohort.** Summary statistics from the published JHU/Netherlands/Germany cohort including all ARVC probands in the registries with a pathogenic or likely pathogenic desmosomal variant (N=322), not only those included here (N=170).^23^

| **gnomAD MAF ≤0.01, OR≥4.5** | | |  |  |  |  |  |
| --- | --- | --- | --- | --- | --- | --- | --- |
| Alpha | N cases | N controls | Window % transcript (% controls exposed) | % cases exposed | OR | window size (bp) | Power |
| 0.01 | 40 | 12408 | 0.1 | 0.45 | 4.5 | 264.3 | 100% |
| 0.01 | 40 | 12408 | 0.0500 | 0.225 | 4.5 | 132.15 | 90% |
| 0.01 | 40 | 12408 | 0.0250 | 0.1125 | 4.5 | 66.075 | 68% |
| 0.01 | 40 | 12408 | 0.0125 | 0.05625 | 4.5 | 33.0375 |  |

| **gnomAD MAF ≤0.001, OR≥4.5** | | |  |  |  |  |  |
| --- | --- | --- | --- | --- | --- | --- | --- |
| Alpha | N cases | N controls | Window % transcript (% controls exposed) | % cases exposed | OR | window size (bp) | Power |
| 0.01 | 40 | 3970 | 0.1 | 0.45 | 4.5 | 264.3 | 100% |
| 0.01 | 40 | 3970 | 0.0500 | 0.225 | 4.5 | 132.15 | 90% |
| 0.01 | 40 | 3970 | 0.0250 | 0.1125 | 4.5 | 66.075 | 67% |
| 0.01 | 40 | 3970 | 0.0125 | 0.05625 | 4.5 | 33.0375 |  |
|  |  |  |  |  |  |  |  |
| **gnomAD MAF ≤3.6X10^-5^,OR≥4.5** | | | |  |  |  |  |
| Alpha | N cases | N controls | Window % transcript (% controls exposed) | % cases exposed | OR | window size (bp) | Power |
| 0.01 | 40 | 1020 | 0.1 | 0.45 | 4.5 | 264.3 | 100% |
| 0.01 | 40 | 1020 | 0.0500 | 0.225 | 4.5 | 132.15 | 88% |
| 0.01 | 40 | 1020 | 0.0250 | 0.1125 | 4.5 | 66.075 | 65% |
| 0.01 | 40 | 1020 | 0.0125 | 0.05625 | 4.5 | 33.0375 |  |

| **gnomAD MAF ≤3.6X10^-5^,OR≥20** | | | |  |  |  |  |
| --- | --- | --- | --- | --- | --- | --- | --- |
| Alpha | N cases | N controls | Window % transcript (% controls exposed) | % cases exposed | OR | window size (bp) | Power |
| 0.01 | 40 | 1020 | 0.1 | 0.45 | 20 | 264.3 | 100% |
| 0.01 | 40 | 1020 | 0.0500 | 0.225 | 20 | 132.15 | 100% |
| 0.01 | 40 | 1020 | 0.0250 | 0.1125 | 20 | 66.075 | 100% |
| 0.01 | 40 | 1020 | 0.0125 | 0.05625 | 20 | 33.0375 | 99% |

**Supplementary Table 3. Power calculations to determine window size based on sample size.** Assuming equal distribution of gnomAD variants across the transcript, % exposed in the control group was set at the percent of the transcript included in a given window size. OR cut off was predetermined as indicated and percent cases exposed calculated based on this and % controls exposed. Only probands carrying variants with population MAF ≤ 3.6X10^-5^  were included (N=40). All statistical analyses represented here were performed at <http://openepi.com/Power/PowerCC.htm>.

| **Window Size** | **gnomAD MAF≤** | **% trans. enriched for ACM** | **Known Functional Domains Captured** | **Odds of ACM in combined enriched windows** | **Etiologic Fraction for ACM among patients with variants in PKP2** | **p value** |
| --- | --- | --- | --- | --- | --- | --- |
| **132 bp** | **0.01** | 50.0 | ARM1-8 | 18.4[9.2,38.3] | 0.94 [0.89,0.97] | 8.17E-34 |
| **132 bp** | **0.001** | 25.4 | ARM 1-4,8 | 7.4[3.8,14.7] | 0.86[0.73,0.93] | 2.25E-13 |
| **132 bp** | **3.6x10^-5^** | 1.2 | none | 11.6[1.9,53.1] | 0.91[0.46,0.98] | 1.35E-05 |
| **33 bp** | **0.01** | 64.9 | HR2  ARM1-8 | 96.5[39.8,281.9] | 0.98[0.97,0.99] | 3.4E-102 |
| **33 bp** | **0.001** | 18.9 | HR2  ARM1-5,8 | 31.4[15.2,68.4] | 0.96[0.93,0.96] | 4.71E-51 |
| **33 bp** | **3.6x10^-5^** | 7.3 | ARM1-3,5,7 | 16.4[7.2,36.24] | 0.94[0.86,0.97] | 4.54E-22 |

**Supplementary Table 4. Rational development of a regional assessment heuristic for missense variants.** For a given window width and population MAF restriction, we show the percent of the transcript lying within ACM-enriched regions, the known functional domains included in these regions, and the etiologic fraction of ACM diagnoses explained by variants in these windows among patients with any variant in *PKP2*. MAF: Minor Allele Frequency. ARM: Armadillo Repeat, HR2: Head domain.

| **Cohort** | **Ethnic/Ancestry Admixture** |
| --- | --- |
| **Invitae/LMM**  *Truncating (N=98)*  European  African/African American  Asian/Asian American  Mixed/Other/Unknown  *Missense (N=40)*  European  African/African American  Asian/Asian American  Mixed/Other/Unknown | 67 (68.4%)  3 (3.1%)  4 (4.1%)  24 (24.5%)  25 (62.5%)  7 (17.5%)  0 (0%)  8 (20%) |
| **gnomAD**  *Truncating (N=97)*  European  African/African American  Asian/Asian American  Other  Mixed  *Missense ((MAF<0.0001), N=1768)*  European  African/African American  Asian/Asian American  Other  Mixed | 26 (26.8%)  6 (6.2%)  15 (15.5%)  8 (8.2%)  42 (43.3%)  255 (14.4%)  114 (6.4%)  296 (16.7%)  90 (5.1%)  1013 (57.3%) |
| **Definite ARVC Cohort**  *International Missense Cohort (N=16)*  European  *JHU/Netherlands ARVC Registry^23^ (N=322)*  European  African/African American  Asian/Asian American | 16 (100%)  314 (97.5%)  3 (0.9%)  5 (1.6%) |
| **MyCode**  *Truncating (N=34)*  European  African/African American  Asian/Asian American  Mixed/Other/Unknown  *Missense ((MAF<0.001), N=1662)*  European  African/African American  Asian/Asian American  Mixed/Other/Unknown | 28 (82%)  6 (18%)  0  0  1370 (82%)  203 (12%)  38 (2%)  51 (3%) |

**Supplementary Table 5. Ethnicity and Ancestry Admixture of Included Cohorts.** Summary statistics from the published JHU/Netherlands/Germany cohort including all ARVC probands in the registries with a pathogenic or likely pathogenic desmosomal variant (N=322), not only those included here (N=170).


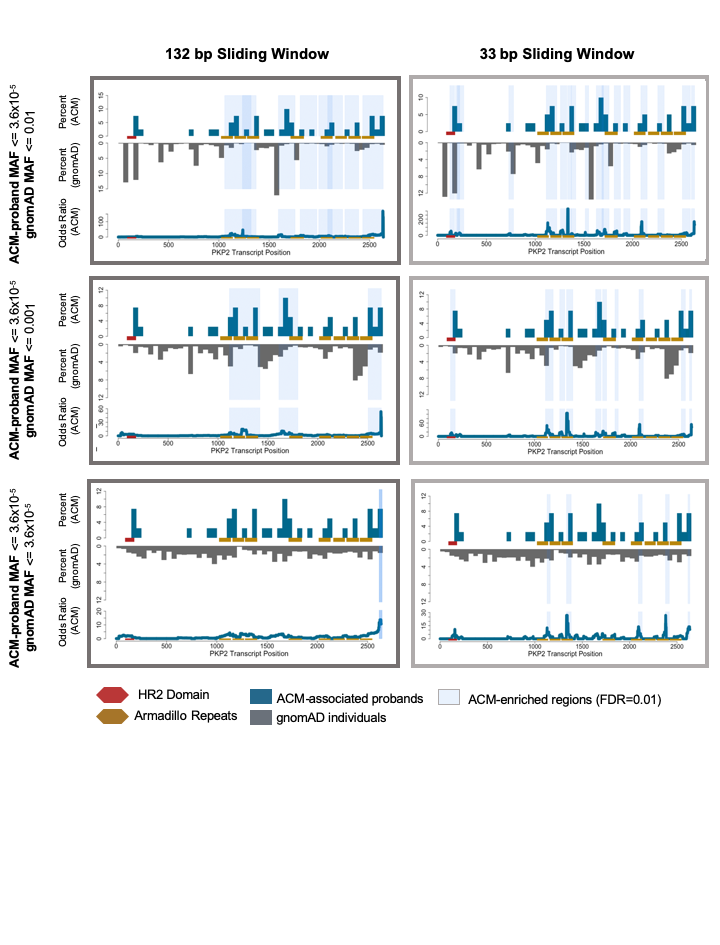
 **Supplementary Figure 1. Rational development of a regional assessment heuristic for missense variants.** Progressive MAF restriction in gnomad and decrease in window size allows for detection of known and previously unrecognized functional domains. The same analysis was performed using an MAF restriction of 0.0001 in gnomAD with a 33 bp sliding window, and detected regions were similar to those detected using the 33 bp window and gnomad MAF restriction of 3.6X10^-5^ with the inclusion of windows from c.1636-1749 identified using the gnomad MAF 0.001 restriction. Regardless of these variables, the C-terminus remains enriched.


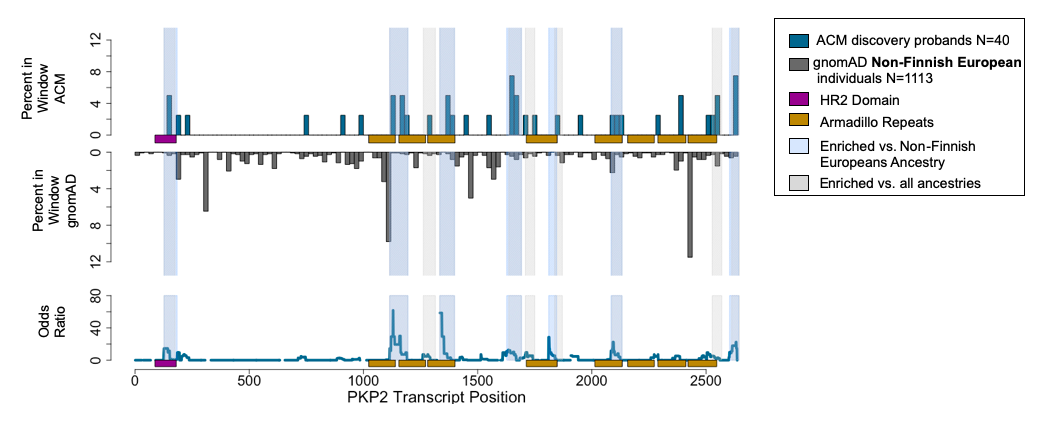
**Supplementary Figure 2. Sensitivity analysis with restriction of gnomAD population to individuals of non-Finnish European ancestry.** This slightly narrows discovery of enriched regions (light blue regions) compared to the use of mixed ancestry controls (grey regions), and does not reveal region around p.C796R founder variant. Notably of the 31 ACM probands included, 18 (58%) were of European and 8 (25%) were of African ancestry. 6% were of Asian Ancestry and 10% were mixed/other. Non-Finnish European individuals limited to variants with MAF≤0.001. ACM probands limited to MAF ≤3.6X10^-5^.

# Supplementary Files

1. List of all included variants in discovery and validation datasets
2. International ARVC Missense Variant Cohort Clinical Characteristics
3. Final odds of disease inclusion by sliding window
